# Supplementary material for: The Construction and Exploration of a Comprehensive MicroRNA Centered Regulatory Network in Foxtail Millet (Setaria italica L.)
Source: Front Plant Sci. 2022 May 6;13:848474. doi: 10.3389/fpls.2022.848474 (PMC9121102; doi:10.3389/fpls.2022.848474)
Supplement: Supplementary file 4 [file Data_Sheet_4.zip › Sit-miR398a_Seita.7G183000.1_395_TPlot.pdf]

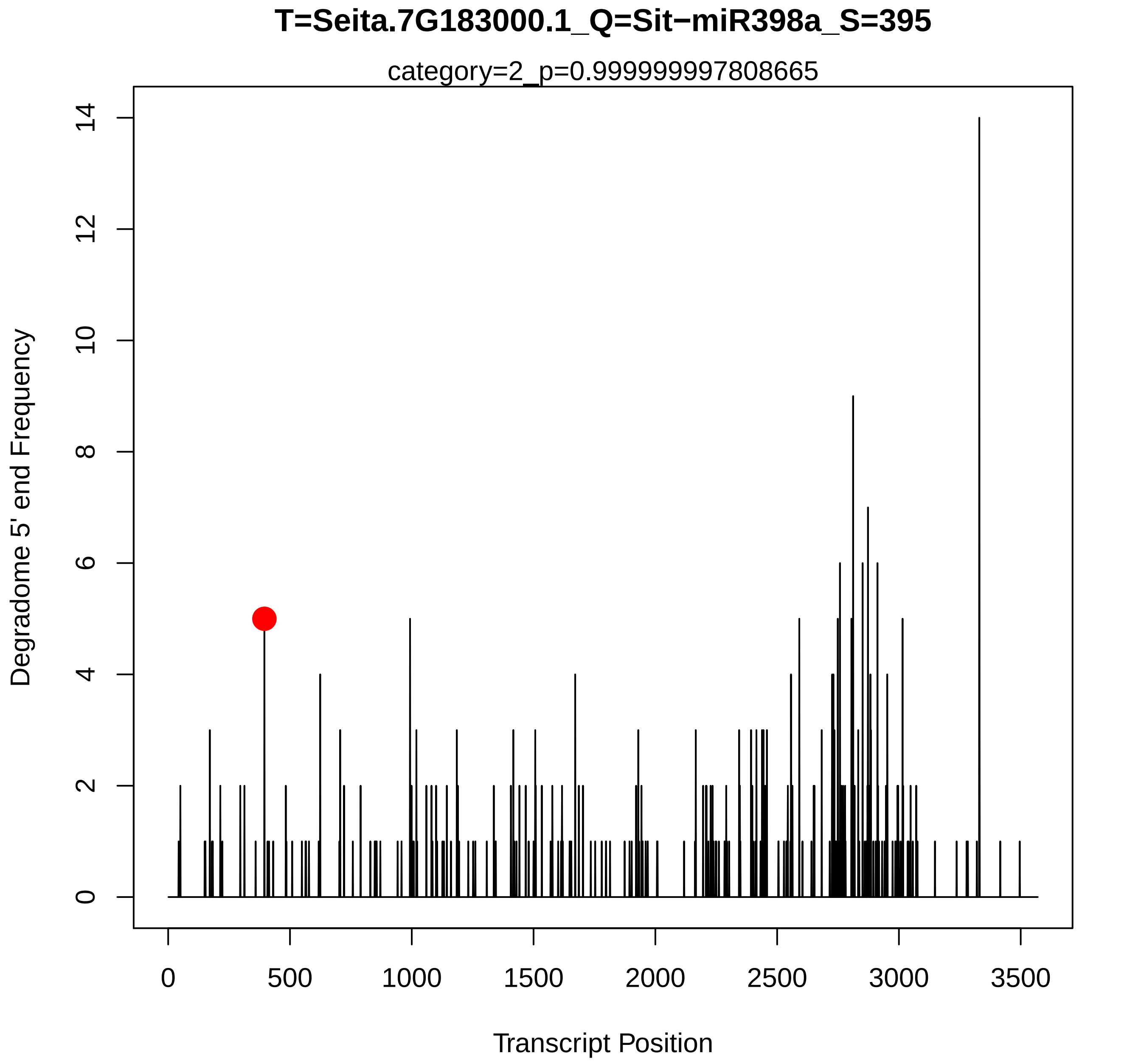

T=Seita.7G183000.1_Q=Sit−miR398a_S=395
category=2_p=0.999999997808665
Degradome 5' end Frequency
Transcript Position
0
2
4
6
8
10
12
14
0
500
1000
1500
2000
2500
3000
3500
